# The experiences of women with breast cancer who undergo fertility preservation

**DOI:** 10.1093/hropen/hoab018

**Published:** 2021-04-29

**Authors:** T Dahhan, F van der Veen, A M E Bos, M Goddijn, E A F Dancet

**Affiliations:** 1 Center of Reproductive Medicine, Amsterdam UMC (location AMC), University of Amsterdam, Amsterdam, The Netherlands; 2 Center of Reproductive Medicine, University Medical Center Utrecht, Utrecht, The Netherlands; 3 Department of Development and Regeneration, Research Foundation—Flanders, University of Leuven, Leuven, Belgium

**Keywords:** cryopreservation, vitrification, assisted reproduction, female infertility, ovarian stimulation, psychology, qualitative research, reproductive decision making, gonadotrophins

## Abstract

**STUDY QUESTION:**

How do women, who have just been diagnosed with breast cancer, experience oocyte or embryo banking?

**SUMMARY ANSWER:**

Fertility preservation was a challenging yet welcome way to take action when confronted with breast cancer.

**WHAT IS KNOWN ALREADY:**

Fertility preservation for women with breast cancer is a way to safeguard future chances of having children. Women who have just been diagnosed with breast cancer report stress, as do women who have to undergo IVF treatment. How women experience the collision of these two stressfull events, has not yet been studied.

**STUDY DESIGN, SIZE, DURATION:**

We performed a multicenter qualitative study with a phenomenological approach including 21 women between March and July 2014. Women were recruited from two university-based fertility clinics.

**PARTICIPANTS/MATERIALS, SETTING, METHODS:**

Women with breast cancer who banked oocytes or embryos 1–15 months before study participation were eligible. We conducted in-depth, face-to-face interviews with 21 women, which was sufficient to reach data saturation.

**MAIN RESULTS AND THE ROLE OF CHANCE:**

The 21 women interviewed had a mean age of 32 years. Analysis of the 21 interviews revealed three main experiences: the burden of fertility preservation, the new identity of a fertility patient and coping with breast cancer through fertility preservation.

**LIMITATIONS, REASONS FOR CAUTION:**

Interviewing women after, rather than during, fertility preservation might have induced recall bias. Translation of quotes was not carried out by a certified translator.

**WIDER IMPLICATIONS OF THE FINDINGS:**

The insights gained from this study of the experiences of women undergoing fertility preservation while being newly diagnosed with breast cancer could be used as a starting point for adapting the routine psychosocial care provided by fertility clinic staff. Future studies are necessary to investigate whether adapting routine psychosocial care improves women’s wellbeing.

**STUDY FUNDING/COMPETING INTEREST(S):**

None of the authors in this study declare potential conflicts of interest. The study was funded by the Center of Reproductive Medicine of the Academic Medical Center.

**WHAT DOES THIS MEAN FOR PATIENTS?**

Women with breast cancer who freeze eggs or embryos are confronted with emotional challenges. This study interviewed 21 women who froze their eggs or embryos while having breast cancer and asked them about their experiences. Women revealed that it was difficult for them to combine having cancer with undergoing IVF treatment, but fertility preservation also offered ways to help them cope with breast cancer.

## Introduction

Fertility preservation offers women with cancer the possibility to safeguard their ability to have children in the future (Martinez 2017; Burns 2018). Yearly about 18.1 million new cases of cancer are reported worldwide, of which breast cancer is the most common malignancy diagnosed in young women (Bray *et al.*, 2018). Breast cancer therapy threatens fertility in different ways. First, the therapy often has gonadotoxic side effects (Meirow and Nugent, 2001; Sukumvanich *et al.*, 2010, Spears *et al.*, 2019). Second, women affected by BRCA 1 or 2 gene mutation can even be offered a bilateral salpingo-ovariectomy to prevent ovarian cancer (Begg *et al.*, 2008). Third, women with cancer are often advised to delay pregnancy because of the risk of recurrence and this delay leads to age-related fertility decline (RCOG, 2011). A pregnancy delay of no <5 years is advised to women with hormone-sensitive breast cancers who are treated with tamoxifen as adjuvant therapy (Barthelmes and Gateley, 2004; Braems *et al.*, 2011).

To deal with these threats to their fertility, women can currently opt to bank oocytes or embryos after controlled ovarian stimulation (Rienzi *et al.*, 2017; Niederberger *et al.*, 2018). Controlled ovarian stimulation and IVF are known to cause distress in subfertile women (Verhaak *et al.*, 2007; Brod and Fennema, 2013). Providing timely and structured information about fertility preservation to women has, therefore, been deemed important for enabling adequate decision-making whilst in the midst of the stressful event of having cancer (Hill *et al.*, 2012; Baysal *et al.*, 2015; Dahhan *et al.*, 2015). Studies showed that breast cancer survivors have specific breast-cancer related worries about childbearing (i.e. fear for hormone-induced relapse of disease during pregnancy) and motherhood (i.e. fear of relapse of disease while having children; Dow, 1994; Connell *et al.*, 2006; Lee *et al.*, 2011; Goncalves *et al.*, 2014).

The experience of having breast cancer and simultaneously undergoing fertility preservation to safeguard a future with children has not yet been studied. Insight into women's experiences and needs during treatment is a pre-requisite for the successful implementation of fertility preservation care (Anazodo *et al*., 2019; Gameiro *et al.*, 2015).

This study aimed to explore how women experience oocyte or embryo banking when they have just been diagnosed with breast cancer.

## Materials and methods

A phenomenological design was chosen as phenomenology is a specific qualitative research methodology devoted to exploring and understanding experiences, including experiences of health care (Polkinghorne, 1989; Giorgi, 2000).

### Ethical approval

The Institutional Review Boards of the Academic Medical Centre (AMC) Amsterdam and of the University Medical Centre Utrecht (UMCU) considered the protocol of this study (W13_212#13.17.0266) and confirmed that participating women would not be subjected to any risks. Therefore, no further review was required according to the Dutch ‘Medical Research Involving Human Subjects Act’. Participating women did provide written informed consent before participation.

### Recruitment of women

All women aged 18–43 years who were newly diagnosed with breast cancer and who banked their oocytes or embryos in the Dutch Centers for Reproductive Medicine of the Amsterdam University Medical Center or the University Medical Center Utrecht between January 2013 and July 2014 were eligible for inclusion. These women received a letter by postal mail that informed them about the aim and the confidential nature of the study and the contact details of the researcher. Women who did not contact the researcher themselves received a telephone call 2 weeks later. The 10 women who had most recently banked their oocytes or embryos were contacted first. Another 18 women were contacted in the second round of recruitment. Recruitment stopped when new data did not yield new insights, meaning that data saturation was achieved (Guest *et al.*, 2006).

### Data collection

After obtaining informed consent, data on demographics (i.e. age, education, ethnicity and relationship status) and on medical background (i.e. date of breast cancer diagnosis, type of tumor and cancer treatment and number of oocytes or embryos retrieved) were collected by means of a questionnaire.

Twenty interviews were conducted by TD (a female medical doctor and PhD student) and one interview by ED (a female midwife with post-doctoral experience and a PhD in fertility care).

Before data collection, TD had written down her preconceived beliefs about women’s experiences in a reflective journal and had discussed these with ED to try to neutralize her role as a co-participant in the in-depth conversation (Lofland and Lofland, 1995). To further increase their neutral role towards the interviewed women, TD and ED were not involved in clinical care at the time of the study.

Depending on women’s preferences, interviews took place at their home (n = 15), at the fertility clinic (n = 5) or at a public place (n = 1).

The face-to-face in-depth interviews, which lasted 45–90 min, were guided by an introductory open-ended question (i.e. ‘How did you experience having breast cancer while freezing oocytes or embryos?’) and by probing questions derived from a topic list based on a literature review. The sequence and formulation of the probing questions depended on the interview situation, resulting in open and in-depth interviews (Weiss, 1994). The interviews were conducted in Dutch. The primary researcher has good knowledge of the English language and was able to translate the text. The interviews were audio-recorded and transcribed verbatim, as this process guarantees that the text consists of the natural language used by the interviewed participant (Wester, 1995). In addition, field notes of important non-verbal communication were taken during the interviews.

### Data analysis

The phenomenological analysis was focused on learning more about the patient’s experience and was supported by MAXQDA software (Max Qualitative Data Analysis, version 11, VERBI Software, Germany). The analysis of the verbatim transcripts was conducted according to the following four steps for inductive analysis: the explorative phase, the specification phase, the reduction phase and the integration phase (Wester, 1995).

During the explorative phase, the interviews were read thoroughly as a whole. During the specification phase, meaningful fragments that somehow answered our research question were labeled by a theme and code, which formed the basis of a coding tree. During the reduction phase, the codes were organized according to their importance and ability to answer the research question, after which the coding tree was reduced. During the integration phase, connections were made between the codes to integrate them into an overall meaning of how the banking of oocytes or embryos was experienced by the interviewed women. Once the coding tree was formed, the interviews were read as a whole again to check for meaningful units of text which could be added to the existing codes or which required adding a new code to the coding tree.

Interviews were organized until no new codes emerged during analysis, which was a process of combining data collection with analysis. More specifically, when redundant and no new information was collected (i.e. data-saturation) and when no new codes emerged during analysis (i.e. inductive thematic saturation) we considered the number of interviewed women to be sufficient to answer the research question (Saunders *et al.*, 2018).

One researcher (TD) analyzed the interviews and discussed the codes, their meaningful units of text and their order in the coding tree with a second researcher (ED) to increase the ‘inter-rater reliability’ or trustworthiness of the data analysis (Boeije, 2005). Discrepancies were discussed until consensus was reached. Data collection and analysis alternated so that emerging new ideas from early data could lead to revising and adjusting the interview topic list (Mays and Pope, 1995).

Throughout the results section, below, interview quotations identified by pseudonyms are provided to describe the phenomenon of the lived experience of women with cancer undergoing fertility preservation.

With respect to reporting the data, we used the consolidated criteria for reporting qualitative research (COREQ), which is comparable to the Consolidated Standards of Reporting Trials (CONSORT; Moher *et al.*, 2001; Tong *et al.*, 2007).

## Results

### The participating women

We invited 28 women of whom 21 consented to participate (participation rate = 75%). Women who declined were on holiday during the interview period (n = 3), considered cancer a ‘closed chapter’ which they did not want to talk about (n = 2) or were interviewed but refused to be recorded (n = 2).

The participating women were aged 32 years on average. At the time of the interview, fertility preservation had, on average, taken place 7.9 months prior. The characteristics of the 21 participating women are presented in Table I. Data saturation was achieved after 18 interviews and confirmed by the last three interviews.

**Table I:** Demographic characteristics, relationship status and medical characteristics of the 21 participating women with breast cancer who banked oocytes or embryos.

Age in years (mean, range)	32 (25–39)
Educational level (n)	
** **University	6
** **University college	11
** **High school education	4
Nationality (n)	
** **Dutch	20
** **Other	1
Stable relationship during fertility preservation (n)	18
Single during fertility preservation (n)	3
Relationship ended during fertility preservation (n)	2
Was attempting pregnancy just before breast cancer diagnosis (n)	6
Had children at time of breast cancer diagnosis (n)	4
Had a hormone receptor-positive breast tumor (n)	14
Had tumor surgically removed before fertility preservation	9
Number of women banking oocytes	15
Banked oocytes (n)	15
Banked embryos (n)	5
Stopped before follicle aspiration	1
Number of oocytes banked per woman (mean, range)	15 (9–27)
Number of embryos banked per woman (mean, range)	7 (1–17)
Could not undergo more than one cycle (n)	15
Time in months between treatment and the interview (mean, range)	7.9 (1–16)

### The lived experience

Analysis of the 21 interviews revealed three main experiences: the burden of fertility preservation, the new identity of a fertility patient and coping with breast cancer through fertility preservation.

Detailed codes for each of these three main experiences are presented in Table II.

**Table II:** Results grouped by the emerged themes of the coding tree.

	Number of women reporting on this experience
**The burden of fertility preservation**	
Stress, as only one cycle of fertility preservation, could be performed to make it in time before starting chemotherapy	12
Fear of having complications of fertility preservation	10
Difficulty distinguishing several emotional side effects	7
Discomfort in undergoing treatment for breast cancer and for fertility preservation in terms of intimacy	6
Traveling between hospitals	4
Stress regarding safety of fertility preservation because of possible hormone-induced tumor growth	4
Insecurity about effectiveness of fertility preservation	3
Disappointment of having a low number of banked oocytes or embryos made starting chemotherapy difficult	2
**The new identity of a fertility patient**	
Feeling like an outsider with regard to regular IVF patients	16
Consequences on partner’s family planning	8
Pre-occupied with possible age-related subfertility because of medical advice to delay pregnancy for a long period	7
Secrecy about fertility preservation	5
Not wanting to belong to the group of patients who have non-cancer-related infertility issues because of *a priori* objections against assisted reproduction	5
Seeing other fertility patients in the waiting room offered relief	3
**Coping with breast cancer through fertility preservation**	
Fertility preservation allowed taking action while having lost control because of the cancer diagnosis	16
Having oocytes or embryos banked gave strength to start chemotherapy	11
Fertility preservation called attention to well-functioning part of body	7
Fertility preservation offered romantic relief in stressful period	7
Fertility preservation as a means to invest in a future as breast cancer survivor	4

#### The burden of fertility preservation

Women shared how difficult it was that their diagnosis of cancer had to co-exist with the threat to their fertility. This notion is well articulated by Wendy. *‘When I was young, I was horrified by the thought that some women had to rely on IVF to have children. I also thought that undergoing chemotherapy because you have cancer would be horrible. Then, on one and the same day, I was confronted with both’*.

Women with hormone-sensitive breast cancer (n = 14) were confronted with the threat of increased hormone levels during ovarian stimulation. These women shared that they experienced a threat of possible cancer growth due to fertility preservation. Coping mechanisms included trying not to let the spectre of cancer growth due to the hormonal sensitivity of the tumor dominate their thoughts. Sandra, another participant had this to say: *‘They said that this medication [for ovarian stimulation] could stimulate the growth of my tumor. Looking back, I was not at all worried about that [***_*…*_***] You have no mental space left to deal with these things, you know*’. In addition, worries about the potential growth of a hormone-sensitive tumor during pregnancy were postponed as exemplified by what Martina said: ‘*Regarding tumors after the pregnancy period [sigh] I will start thinking about that when I have a partner with whom I have a stable life’.*

Fertility preservation coincided with the breast cancer treatment trajectory of all interviewed women: they visited the fertility clinic while having started radiotherapy, while recovering from breast surgery and/or while consulting their oncologist to receive test results related to breast cancer staging or genetic mutations. There was no firm consensus as to whether the cancer treatment trajectories overshadowed fertility preservation or the other way around. For example, Kiara said: *‘I regard my IVF treatment as a tiny branch of my breast cancer treatment’.* Nika said: *‘I really wanted to do it [fertility preservation] right, so I decided to focus on IVF and once that was finished I would continue thinking about radiotherapy and chemo. I completely blocked thoughts about chemotherapy to undergo IVF first’*. The intertwined treatment trajectories made it difficult for women to say which emotions were caused by which treatment, Yara said: *‘When I felt emotional, I wondered whether this was because of the IVF medication or because of everything else I had to go through’.*

Requiring breast cancer treatment shortly after fertility preservation resulted in an intense time pressure during fertility preservation. As a result, Brenda states, *‘I was very nervous and every time [I injected myself] I wondered whether I did it correctly. So much depended on it, you know. You can only do it right one time. It made me feel very insecure’*. The time pressure even made Vivienne decide to cancel her treatment: ‘*I really wanted it, but it just didn’t work because only about 6-7 eggs grew [.]. I thought I needed at least 30, so six is not enough. So I thought, you know what, I quit’*.

Finally, women felt uncomfortable revealing private body parts during their breast cancer treatment and during fertility preservation. Martina said: *‘During one treatment you have to be naked from the waist up, during the other treatment you have to be naked from the waist down. That’s a very unpleasant experience’*.

#### The new identity of a fertility patient

Women were confronted with two new identities: the identity of a ‘cancer patient’ and of a ‘fertility patient’. The identity of a ‘fertility patient’ was unpleasant for some women. Women were anxious about the reactions from their social environment. Martha, for example, described the moment when she got her IVF medication from a pharmacy*: ‘I noticed that people were staring at me and I could almost hear them thinking “what could her problem be”—after they overheard the word ‘IVF’. It made me feel very insecure, because with breast cancer you already feel like you’re in the spotlight [***_*…*_***] you are very aware of yourself, I mean, I was walking around with my cotton prosthesis and you don’t want people to see that you are missing your breast’.*

Also, women described feelings of shame. Sue said: ‘*Almost no one knew I was doing this [fertility preservation]. I thought they would judge me for undergoing this treatment. You already have two children and you have breast cancer, they might think: “what are you doing to yourself?” ’*.

Women were ambivalent about whether or not they belonged to the bigger group of ‘regular’ fertility patients. For some women, this was a group they never thought they would belong to. Mona said: *‘Before [breast cancer] I thought if it happens the natural way that’s fine, but I will not go through all those procedures because I urgently want to have children. And now, I am confined to these procedures. When I was at the fertility clinic, I realized that I, all of a sudden, became part of this group of people who rely on IVF to have children’*.

Women were relieved that they were not the only ones relying on medically assisted reproduction, as explained by Hanna: *‘I was surprised how busy it was in the waiting room. That comforted me somehow. I thought, I may have breast cancer but there are plenty of people with other reasons for whom having children may be difficult’*.

Women wondered whether they were worse or better off than ‘regular’ fertility patients, ‘*You see all these couples [***_*…*_***] and I felt different from them. I thought:* ***“****Guess why I’m here***_*…*_***breast cancer! [.] maybe I was a bit jealous of them, as I’d rather be in their situation’, Hanna said.* Sandra on the other hand explains that *‘You appreciate the things that function well [ovarian function]. I was in the middle of people having problems with fertility, which was something that I did not have a problem with at that moment [at time of fertility preservation]. I was fertile because I had not yet undergone chemotherapy. That made me feel very good’*.

#### Coping with breast cancer through fertility preservation

Their new breast cancer diagnosis made women anxious about their future and yet undergoing fertility preservation gave them a new prospect for their future, on which they could focus. Martha said: *‘They often say “you have to fight against cancer”, but I prefer saying “you should fight FOR something”. I fought for having children.* Hanna said: *‘[by going through fertility preservation] I was working on my future and thought, “[my life] doesn’t have to end”. I had a very positive perspective for the future, otherwise they [the oncologists and reproductive gynecologist team] wouldn’t have offered me this procedure’.*

For some women, even, fertility preservation was a romantic experience. Ikram said: *‘Me and my husband made a picture of us together during ovum pick-up. We wanted to capture that moment as a memory for our future children’.*

Women’s relationship with their cancer identity brought about a strong survival mode, which in turn helped them cope with the burden of fertility preservation. Martha said: *‘I told myself explicitly “keep your survival-mode on”. I need my survival mode because it enables me to make good and rational choices*’.

## Discussion

This is the first phenomenological study to provide an in-depth insight into the lived experience of fertility preservation right after being diagnosed with cancer, which is complementary to the available knowledge about fertility preservation counseling (Hoeg *et al.*, 2016). Combining the burden of being a fertility patient with that of being a patient with cancer was challenging for women. Fertility preservation was, nevertheless, a welcome way to take action when just confronted with having breast cancer.

Our phenomenological approach allowed enhancing clinicians’ understanding of the ‘phenomenon’ of fertility preservation right after being diagnosed with cancer through the eyes of those who are experiencing it (Patton, 2002; Creswell and Poth, 2018). We optimized the quality of our reporting on this qualitative study by relying on the consolidated criteria for reporting qualitative research (COREQ,) which is comparable to the Consolidated Standards of Reporting Trials (CONSORT; Moher *et al.*, 2001; Tong *et al.*, 2007).

We used several strategies to safeguard the trustworthiness of our qualitative data collection and analysis. We enhanced the credibility of our study by including a diverse sample, and interviewing individuals (rather than groups) in the location of their preference (i.e. clinic and public place) on the sensitive topics of cancer and fertility. In addition, we returned our results to the participants to check for accuracy and resonance with their experiences (i.e. member checking). Finally, the main researcher wrote down prior assumptions and kept a reflective diary to prevent personal and intellectual biases, and to enhance the credibility of the findings (Mays and Pope, 1995). The dependability of the study was safeguarded by always using the same open-ended question and topic list, and by regular discussions among the researchers during the intertwined processes of data collection and analysis (Thomas and Magilvy, 2011).

Only one researcher coded the interviews but discussed all phases of the analysis with a second researcher until consensus was reached. This increased the dependability of the analysis. To allow the reader to judge the transferability of our findings, our methodology and sample were described in detail and interview quotations are provided. We acknowledge that the translation of the interviews was not carried out by a certified translator. Interviewing women after, rather than during, fertility preservation might have induced recall bias. We decided to interview the women after fertility preservation because women can be in a state of shock immediately after hearing their diagnosis, which might mask their ability to reflect on their experiences (Taylor, 2000; Landmark *et al.*, 2001). Our findings suggest that recall bias was limited as women who underwent fertility preservation <2 months before the interview reported similar experiences as women for whom fertility preservation had taken place >2 months earlier—a randomly chosen time frame that seemed appropriate to define a more recent treatment.

Our study confirms the previous findings that women with cancer value their fertility and that offering fertility preservation helps women feel in control and helps them believe in a life after cancer (Ehrbar *et al.*, 2016; Hoeg *et al.*, 2016). Our study found that women consider fertility preservation an integrated part of their breast cancer treatment trajectory and that it offered women a prospect for their future. This finding differs from findings in other studies that show that women consider fertility concerns secondary to the importance of survival (Gorman *et al.*, 2011; Lee *et al.*, 2011).

Our findings underpin the relevance of the advice of the American Society of Clinical Oncology to considered fertility preservation as an integral part of women’s breast cancer treatment trajectory (Oktay *et al.*, 2018). The identified ‘survival mode’ which induces an eagerness to act by preserving fertility and a tendency to push emotions aside is in line with previous studies reporting that women with newly diagnosed breast cancer use hope and looking forward as a coping strategy (Taylor, 2000; Landmark *et al.*, 2001).

Although fertility preservation in itself allowed the women in our study to take action and have a future prospect of bearing children, the medical procedures undertaken combined with being viewed by others as an infertility patient and a cancer patient was challenging for women.

Women’s worries about injecting hormones while having a hormone-sensitive tumor confirm the clinical problem caused by the lack of evidence on the safety of controlled ovarian stimulation in terms of the prognosis of breast cancer (Dahhan *et al.*, 2013; Anderson *et al.*, 2020).

Women were also occupied with the threat to their fertility because of the advice to delay pregnancy for at least 2 years. This suggests that women with breast cancer also consider themselves at risk for age-related subfertility, which can easily be overlooked by clinicians dichotomizing indications for fertility preservation into medical and non-medical reasons.

Patients who have non-cancer-related infertility issues have reported stress and anxiety during their treatment as a result of their insecurity on whether they will get pregnant, the treatment burden of having to inject medication and the interference of treatment with their daily life (Verhaak *et al.*, 2007; Brod and Fennema, 2013). Women with breast cancer experienced different emotions. They experienced their treatment in the fertility clinic as a way to help them deal with breast cancer since it reassured them that their reproductive organs were still functioning well.

In conclusion, our data on the experiences of women with breast cancer undergoing fertility preservation can be used to increase clinicians’ understanding and empathy for, and psychosocial care of, these women. Future studies are necessary to investigate ways to incorporate these findings into routine psychosocial care, and to measure its effect on women's wellbeing.

## Data availability

Because of the nature of the collected data (personal interviews), the data will not be shared publicly to protect the privacy of the women who participated in the study.
